# Racemic *N*-methyl-4-[2-(methyl­sulfan­yl)­pyrimidin-4-yl]-1-(tetra­hydro­furan-3-yl)-1*H*-pyrazol-5-amine

**DOI:** 10.1107/S1600536809014317

**Published:** 2009-04-22

**Authors:** Zhengyu Liu, Kevin K.-C. Liu, Arnold L. Rheingold, Antonio DiPasquale, Alex Yanovsky

**Affiliations:** a Pfizer Global Research and Development, La Jolla Laboratories, 10770 Science Center Drive, San Diego, CA 92121, USA; b Department of Chemistry and Biochemistry, University of California, San Diego, 9500 Gilman Drive, La Jolla, CA 92093, USA

## Abstract

The title compound, C_13_H_17_N_5_OS, was obtained by cyclo­addition of 2-[2-(methyl­sulfan­yl)pyrimidin-4-yl]-3-oxo­propane­nitrile with (tetra­hydro­furan-3-yl)hydrazine dihydro­chloride and subsequent *N*-methyl­ation of 4-[2-(methyl­sulfan­yl)­pyrimidin-4-yl]-1-(tetra­hydro­furan-2-yl)-1*H*-pyrazol-5-amine with methyl iodide. The two mol­ecules in the asymmetric unit have opposite absolute configurations and are related by a noncrystallographic inversion center. Both feature intra­mol­ecular N—H⋯N hydrogen bonds. The geometry of the mol­ecules is similar to that observed in the structure of a single enanti­omer of the title compound.

## Related literature

For the structure of the *R*-enanti­omer component of the racemic title compound, see: Liu *et al.* (2009*a* ▶). For details of the synthesis of the title compound, see: Liu *et al.* (2009*a* ▶,*b* ▶).
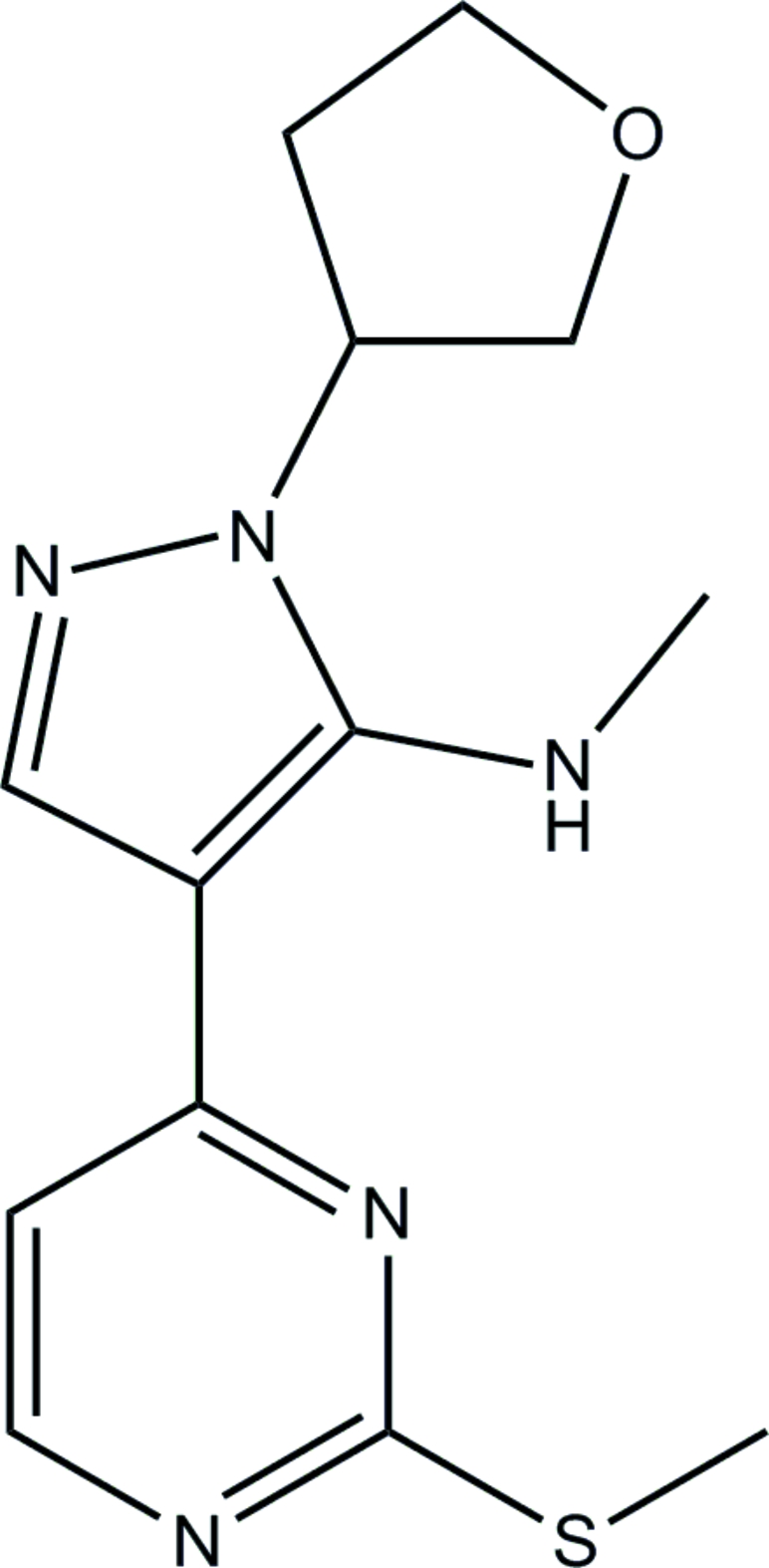


## Experimental

### 

#### Crystal data

C_13_H_17_N_5_OS
*M* *_r_* = 291.38
Monoclinic, 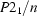

*a* = 15.7404 (5) Å
*b* = 10.1515 (3) Å
*c* = 18.9644 (6) Å
β = 112.829 (1)°
*V* = 2792.92 (15) Å^3^
*Z* = 8
Cu *K*α radiation
μ = 2.10 mm^−1^
*T* = 100 K
0.30 × 0.20 × 0.20 mm

#### Data collection

Bruker *P*4/APEX CCD diffractometer
Absorption correction: multi-scan (*SADABS*; Bruker, 2001 ▶) *T*_min_ = 0.572, *T*_max_ = 0.679
18618 measured reflections
5007 independent reflections
4521 reflections with *I* > 2σ(*I*)
*R*_int_ = 0.030

#### Refinement

*R*[*F* ^2^ > 2σ(*F* ^2^)] = 0.043
*wR*(*F* ^2^) = 0.119
*S* = 1.05
5007 reflections
371 parameters
H atoms treated by a mixture of independent and constrained refinement
Δρ_max_ = 0.88 e Å^−3^
Δρ_min_ = −0.32 e Å^−3^

### 

Data collection: *SMART* (Bruker, 1997 ▶); cell refinement: *SAINT* (Bruker, 1997 ▶); data reduction: *SAINT*; program(s) used to solve structure: *SIR97* (Altomare *et al.*, 1999 ▶); program(s) used to refine structure: *SHELXL97* (Sheldrick, 2008 ▶); molecular graphics: *ORTEP-32* (Farrugia, 1997 ▶); software used to prepare material for publication: *WinGX* (Farrugia, 1999 ▶).

## Supplementary Material

Crystal structure: contains datablocks global, I. DOI: 10.1107/S1600536809014317/dn2448sup1.cif

Structure factors: contains datablocks I. DOI: 10.1107/S1600536809014317/dn2448Isup2.hkl

Additional supplementary materials:  crystallographic information; 3D view; checkCIF report

## Figures and Tables

**Table 1:** Hydrogen-bond geometry (Å, °)

*D*—H⋯*A*	*D*—H	H⋯*A*	*D*⋯*A*	*D*—H⋯*A*
N3—H3N⋯N5	0.82 (2)	2.16 (2)	2.828 (2)	139 (2)
N8—H8N⋯N10	0.83 (2)	2.13 (2)	2.820 (2)	140 (2)

**Fig. 1.**
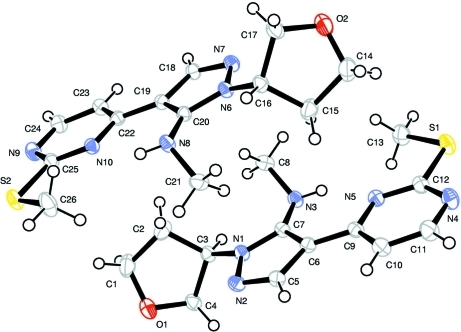
Two independent molecules in the structure of the title compound with the atom labeling scheme. The displacement ellipsoids are drawn at the 50% probability level; H atoms are represented as circles with arbitrary small radius.

## supplementary crystallographic information

### Comment

The title compound was obtained by cycloaddition of 2-(2-(methylsulfanyl)pyrimidin-4-yl)-3-oxopropanenitrile with (tetrahydrofuran-3-yl)hydrazine dihydrochloride and subsequent N-methylation of 4-(2-(methylsulfanyl)pyrimidin-4-yl) -1-(tetrahydrofuran-2-yl)-1H-pyrazol-5-amine with methyl iodide. As cycloaddition may potentially yield one of the two isomeric products differing in the position of the tetrahydrofuranyl substituent, present study was undertaken to establish which of the isomers is actually formed. The X-ray study showed that the product represents the title compound with amino and tetrahydrofuranyl substituents at the neighbouring atoms of the pyrazolyl ring.

There are two molecules in the asymmetric unit (Fig. 1). The molecules are chemically and conformationally identical, but have opposite absolute configurations; moreover, the structure provides an interesting case of almost precise, though non-crystallographic inversion symmetry. The local inversion center has approximate coordinates of -0.001, 0.134, 0.249. Such arrangement may be facilitated by the weak interactions between the H atoms of methyl groups C8 and C21 and the π-electron densities of pyrazolyl rings N6-N7-C18-C19-C20 and N1-N2-C5-C6-C7 respectively: the distances between the methyl C atoms and the centroids of the corresponding rings are 3.520 Å and 3.589 Å.

The geometry of the molecules is very similar to that observed in the structure of single enantiomer obtained by chiral separation of the title compound (Liu *et al.*, 2009a). The methylsulfanylpyrimidine group and pyrazolyl ring lie approximately in one plane; maximum deviations of the C10 and C18 atoms in each of the two molecules are 0.033 (2) Å and 0.037 (2) Å respectively; displacements of methyl C8 and C21 atoms are 0.967 (2) Å and 1.020 (2) Å. Orientation of the tetrahydrofuran ring can be characterized by the dihedral angles 75.6 (1)° and 77.8 (1) formed by the pyrimidine-pyrazolyl planes with the C2-C3-C4 and C15-C16-C17 planes in each of the two molecules respectively.

The secondary amino groups in both molecules (N3 and N8) form intramolecular H-bonds with the N atoms of the pyrimidine rings (N5 and N10 respectively; Table 2).

### Experimental

The detailed descriptions of the synthesis of the title compound are given in Liu *et al.* (2009*a*) and Liu *et al.* (2009*b*).

### Refinement

All H atoms bonded to C atoms were placed in geometrically calculated positions (C—H 0.95 Å, 0.98 Å, 0.99 Å, and 1.00 Å for aromatic, methyl, methylene and methyne H atoms respectively) and included in the refinement in riding motion approximation. The H3N and H8N atoms were located in the difference Fourier map and refined isotropically [N3—H3N 0.82 (2) Å; N8—H8N 0.83 (2) Å]. The *U*_iso_(H) were set to 1.2*U*_eq_ of the carrying atom for non-methyl and amine, and 1.5*U*_eq_ for methyl H atoms.

### Crystal data

**Table:**

C_13_H_17_N_5_OS	*F*(000) = 1232
*M**_r_* = 291.38	*D*_x_ = 1.386 Mg m^−^^3^
Monoclinic, *P*2_1_/*n*	Cu *K*α radiation, λ = 1.54178 Å
Hall symbol: -P 2yn	Cell parameters from 1033 reflections
*a* = 15.7404 (5) Å	θ = 4.9–54.9°
*b* = 10.1515 (3) Å	µ = 2.10 mm^−^^1^
*c* = 18.9644 (6) Å	*T* = 100 K
β = 112.829 (1)°	Block, colorless
*V* = 2792.92 (15) Å^3^	0.30 × 0.20 × 0.20 mm
*Z* = 8	

### Data collection

**Table:**

Bruker P4/APEX CCD diffractometer	5007 independent reflections
Radiation source: fine-focus sealed tube	4521 reflections with *I* > 2σ(*I*)
graphite	*R*_int_ = 0.030
ω scans	θ_max_ = 68.6°, θ_min_ = 3.1°
Absorption correction: multi-scan (*SADABS*; Bruker, 2001)	*h* = −18→18
*T*_min_ = 0.572, *T*_max_ = 0.679	*k* = −11→12
18618 measured reflections	*l* = −22→21

### Refinement

**Table:**

Refinement on *F*^2^	Primary atom site location: structure-invariant direct methods
Least-squares matrix: full	Secondary atom site location: difference Fourier map
*R*[*F*^2^ > 2σ(*F*^2^)] = 0.043	Hydrogen site location: inferred from neighbouring sites
*wR*(*F*^2^) = 0.119	H atoms treated by a mixture of independent and constrained refinement
*S* = 1.05	*w* = 1/[σ^2^(*F*_o_^2^) + (0.0596*P*)^2^ + 1.859*P*] where *P* = (*F*_o_^2^ + 2*F*_c_^2^)/3
5007 reflections	(Δ/σ)_max_ = 0.001
371 parameters	Δρ_max_ = 0.88 e Å^−^^3^
0 restraints	Δρ_min_ = −0.32 e Å^−^^3^

### Special details

**Table:**

Geometry. All e.s.d.'s (except the e.s.d. in the dihedral angle between two l.s. planes) are estimated using the full covariance matrix. The cell e.s.d.'s are taken into account individually in the estimation of e.s.d.'s in distances, angles and torsion angles; correlations between e.s.d.'s in cell parameters are only used when they are defined by crystal symmetry. An approximate (isotropic) treatment of cell e.s.d.'s is used for estimating e.s.d.'s involving l.s. planes.
Refinement. Refinement of *F*^2^ against ALL reflections. The weighted *R*-factor *wR* and goodness of fit *S* are based on *F*^2^, conventional *R*-factors *R* are based on *F*, with *F* set to zero for negative *F*^2^. The threshold expression of *F*^2^ > σ(*F*^2^) is used only for calculating *R*-factors(gt) *etc*. and is not relevant to the choice of reflections for refinement. *R*-factors based on *F*^2^ are statistically about twice as large as those based on *F*, and *R*- factors based on ALL data will be even larger.

### Fractional atomic coordinates and isotropic or equivalent isotropic displacement parameters (Å^2^)

**Table:**

	*x*	*y*	*z*	*U*_iso_*/*U*_eq_	
C1	0.22461 (16)	−0.1765 (2)	0.31756 (13)	0.0393 (5)	
H1A	0.1969	−0.2521	0.2834	0.047*	
H1B	0.2861	−0.1583	0.3170	0.047*	
C2	0.16265 (14)	−0.0551 (2)	0.29178 (11)	0.0310 (4)	
H2A	0.0969	−0.0805	0.2670	0.037*	
H2B	0.1794	−0.0008	0.2558	0.037*	
C3	0.18197 (12)	0.01907 (18)	0.36747 (10)	0.0213 (4)	
H3	0.2191	0.0998	0.3691	0.026*	
C4	0.24143 (13)	−0.07910 (18)	0.42835 (11)	0.0249 (4)	
H4A	0.3068	−0.0511	0.4489	0.030*	
H4B	0.2204	−0.0836	0.4712	0.030*	
C5	−0.01506 (12)	0.02935 (18)	0.41206 (10)	0.0208 (4)	
H5	−0.0595	−0.0101	0.4279	0.025*	
C6	−0.01006 (12)	0.16645 (17)	0.40075 (10)	0.0199 (4)	
C7	0.06411 (12)	0.17913 (17)	0.37729 (10)	0.0196 (4)	
C8	0.11656 (13)	0.29925 (19)	0.28860 (11)	0.0263 (4)	
H8A	0.1802	0.2726	0.2992	0.039*	
H8B	0.1069	0.3899	0.2693	0.039*	
H8C	0.0740	0.2402	0.2501	0.039*	
C9	−0.06565 (12)	0.27261 (18)	0.41090 (10)	0.0203 (4)	
C10	−0.13890 (13)	0.25167 (19)	0.43403 (11)	0.0256 (4)	
H10	−0.1566	0.1654	0.4423	0.031*	
C11	−0.18401 (13)	0.3611 (2)	0.44425 (11)	0.0289 (4)	
H11	−0.2330	0.3485	0.4611	0.035*	
C12	−0.09392 (12)	0.49396 (18)	0.40780 (10)	0.0231 (4)	
C13	0.02328 (15)	0.6340 (2)	0.35791 (13)	0.0331 (5)	
H13A	0.0731	0.5852	0.3971	0.050*	
H13B	0.0462	0.7201	0.3499	0.050*	
H13C	0.0015	0.5842	0.3099	0.050*	
C14	−0.23088 (16)	0.4429 (2)	0.17690 (13)	0.0387 (5)	
H14A	−0.2920	0.4220	0.1775	0.046*	
H14B	−0.2054	0.5207	0.2098	0.046*	
C15	−0.16593 (14)	0.3254 (2)	0.20499 (11)	0.0324 (5)	
H15A	−0.1816	0.2714	0.2417	0.039*	
H15B	−0.1009	0.3544	0.2295	0.039*	
C16	−0.18296 (12)	0.24857 (18)	0.13061 (10)	0.0220 (4)	
H16	−0.2189	0.1669	0.1297	0.026*	
C17	−0.24264 (13)	0.34274 (19)	0.06774 (11)	0.0271 (4)	
H17A	−0.2190	0.3484	0.0265	0.033*	
H17B	−0.3071	0.3110	0.0454	0.033*	
C18	0.01589 (12)	0.24134 (17)	0.08857 (10)	0.0204 (4)	
H18	0.0601	0.2813	0.0726	0.025*	
C19	0.01176 (12)	0.10435 (17)	0.10037 (10)	0.0191 (4)	
C20	−0.06290 (12)	0.09089 (17)	0.12278 (9)	0.0188 (3)	
C21	−0.11957 (13)	−0.03136 (19)	0.20869 (11)	0.0262 (4)	
H21A	−0.0808	0.0312	0.2471	0.039*	
H21B	−0.1069	−0.1210	0.2293	0.039*	
H21C	−0.1847	−0.0103	0.1958	0.039*	
C22	0.06828 (11)	−0.00076 (18)	0.09110 (9)	0.0193 (4)	
C23	0.14461 (12)	0.02003 (18)	0.07200 (10)	0.0224 (4)	
H23	0.1631	0.1063	0.0646	0.027*	
C24	0.19188 (12)	−0.08969 (19)	0.06428 (11)	0.0247 (4)	
H24	0.2430	−0.0772	0.0502	0.030*	
C25	0.09580 (12)	−0.22284 (18)	0.09390 (10)	0.0216 (4)	
C26	−0.03578 (16)	−0.3636 (2)	0.12571 (14)	0.0366 (5)	
H26A	−0.0215	−0.3133	0.1730	0.055*	
H26B	−0.0608	−0.4498	0.1308	0.055*	
H26C	−0.0814	−0.3155	0.0829	0.055*	
N1	0.09784 (10)	0.05689 (15)	0.37756 (8)	0.0199 (3)	
N2	0.04867 (10)	−0.03777 (15)	0.39826 (8)	0.0217 (3)	
N3	0.09942 (11)	0.29183 (15)	0.35925 (9)	0.0220 (3)	
N4	−0.16316 (11)	0.48479 (16)	0.43195 (10)	0.0283 (4)	
N5	−0.04410 (10)	0.39615 (15)	0.39708 (8)	0.0212 (3)	
N6	−0.09717 (10)	0.21269 (14)	0.12244 (8)	0.0197 (3)	
N7	−0.04837 (10)	0.30785 (14)	0.10218 (8)	0.0215 (3)	
N8	−0.09920 (11)	−0.02230 (15)	0.13985 (9)	0.0215 (3)	
N9	0.16958 (10)	−0.21327 (16)	0.07561 (9)	0.0249 (3)	
N10	0.04399 (10)	−0.12520 (15)	0.10180 (8)	0.0200 (3)	
O1	0.23183 (11)	−0.20351 (14)	0.39248 (9)	0.0365 (4)	
O2	−0.23835 (12)	0.46761 (15)	0.10208 (9)	0.0398 (4)	
S1	−0.07026 (3)	0.65654 (5)	0.38885 (3)	0.02884 (14)	
S2	0.06756 (3)	−0.38593 (4)	0.10801 (3)	0.02817 (14)	
H3N	0.0715 (16)	0.355 (2)	0.3657 (13)	0.034*	
H8N	−0.0682 (16)	−0.085 (2)	0.1349 (13)	0.034*	

### Atomic displacement parameters (Å^2^)

**Table:**

	*U*^11^	*U*^22^	*U*^33^	*U*^12^	*U*^13^	*U*^23^
C1	0.0401 (12)	0.0408 (13)	0.0374 (12)	0.0094 (10)	0.0153 (9)	−0.0107 (10)
C2	0.0277 (10)	0.0418 (12)	0.0246 (10)	0.0030 (9)	0.0113 (8)	−0.0049 (8)
C3	0.0195 (8)	0.0206 (9)	0.0254 (9)	0.0011 (7)	0.0106 (7)	0.0006 (7)
C4	0.0231 (9)	0.0236 (9)	0.0296 (10)	0.0047 (7)	0.0120 (8)	0.0033 (7)
C5	0.0199 (8)	0.0209 (9)	0.0222 (9)	−0.0021 (7)	0.0087 (7)	0.0011 (7)
C6	0.0193 (8)	0.0195 (9)	0.0207 (8)	−0.0008 (7)	0.0075 (7)	0.0006 (7)
C7	0.0184 (8)	0.0184 (8)	0.0209 (8)	−0.0011 (7)	0.0063 (7)	−0.0005 (7)
C8	0.0240 (9)	0.0270 (10)	0.0272 (9)	0.0014 (8)	0.0091 (7)	0.0066 (8)
C9	0.0183 (8)	0.0200 (9)	0.0205 (8)	0.0001 (7)	0.0054 (7)	0.0005 (7)
C10	0.0228 (9)	0.0240 (10)	0.0313 (10)	−0.0004 (7)	0.0120 (8)	0.0028 (7)
C11	0.0230 (9)	0.0306 (10)	0.0366 (11)	0.0020 (8)	0.0153 (8)	0.0025 (8)
C12	0.0199 (8)	0.0225 (9)	0.0239 (9)	0.0006 (7)	0.0051 (7)	−0.0018 (7)
C13	0.0408 (11)	0.0218 (10)	0.0438 (12)	−0.0044 (8)	0.0242 (10)	−0.0009 (8)
C14	0.0423 (12)	0.0363 (12)	0.0386 (12)	0.0071 (10)	0.0168 (10)	−0.0073 (9)
C15	0.0290 (10)	0.0423 (12)	0.0265 (10)	0.0078 (9)	0.0114 (8)	−0.0052 (9)
C16	0.0209 (8)	0.0214 (9)	0.0257 (9)	0.0022 (7)	0.0113 (7)	0.0010 (7)
C17	0.0253 (9)	0.0277 (10)	0.0298 (10)	0.0070 (8)	0.0123 (8)	0.0044 (8)
C18	0.0207 (8)	0.0178 (9)	0.0235 (9)	−0.0015 (7)	0.0092 (7)	−0.0009 (7)
C19	0.0191 (8)	0.0180 (9)	0.0195 (8)	−0.0013 (7)	0.0066 (7)	−0.0007 (6)
C20	0.0195 (8)	0.0166 (8)	0.0188 (8)	−0.0008 (7)	0.0057 (6)	−0.0006 (6)
C21	0.0281 (9)	0.0249 (10)	0.0263 (9)	0.0018 (8)	0.0112 (8)	0.0062 (7)
C22	0.0177 (8)	0.0204 (9)	0.0172 (8)	−0.0002 (7)	0.0040 (6)	−0.0005 (6)
C23	0.0201 (8)	0.0209 (9)	0.0252 (9)	−0.0019 (7)	0.0078 (7)	0.0000 (7)
C24	0.0174 (8)	0.0267 (10)	0.0295 (10)	0.0019 (7)	0.0084 (7)	−0.0005 (7)
C25	0.0213 (8)	0.0195 (9)	0.0217 (9)	0.0019 (7)	0.0057 (7)	−0.0009 (7)
C26	0.0449 (12)	0.0194 (10)	0.0575 (14)	−0.0029 (9)	0.0329 (11)	0.0000 (9)
N1	0.0189 (7)	0.0181 (7)	0.0243 (7)	−0.0004 (6)	0.0102 (6)	0.0012 (6)
N2	0.0231 (7)	0.0177 (7)	0.0251 (8)	−0.0026 (6)	0.0101 (6)	0.0021 (6)
N3	0.0229 (8)	0.0170 (7)	0.0292 (8)	−0.0001 (6)	0.0134 (6)	0.0018 (6)
N4	0.0225 (8)	0.0264 (9)	0.0378 (9)	0.0030 (6)	0.0136 (7)	−0.0009 (7)
N5	0.0189 (7)	0.0209 (8)	0.0217 (7)	−0.0006 (6)	0.0058 (6)	−0.0010 (6)
N6	0.0205 (7)	0.0166 (7)	0.0235 (7)	0.0006 (6)	0.0101 (6)	0.0010 (6)
N7	0.0225 (7)	0.0172 (7)	0.0251 (8)	−0.0016 (6)	0.0098 (6)	0.0006 (6)
N8	0.0235 (8)	0.0160 (7)	0.0282 (8)	0.0016 (6)	0.0136 (6)	0.0020 (6)
N9	0.0192 (7)	0.0231 (8)	0.0311 (8)	0.0031 (6)	0.0085 (6)	−0.0017 (6)
N10	0.0201 (7)	0.0180 (7)	0.0204 (7)	−0.0002 (6)	0.0063 (6)	−0.0011 (6)
O1	0.0445 (9)	0.0246 (7)	0.0426 (8)	0.0084 (6)	0.0194 (7)	−0.0002 (6)
O2	0.0538 (10)	0.0272 (8)	0.0448 (9)	0.0133 (7)	0.0260 (8)	0.0044 (6)
S1	0.0310 (3)	0.0180 (2)	0.0391 (3)	0.00095 (18)	0.0152 (2)	−0.00141 (18)
S2	0.0329 (3)	0.0160 (2)	0.0385 (3)	0.00297 (18)	0.0170 (2)	0.00048 (18)

### Geometric parameters (Å, °)

**Table:**

C1—O1	1.408 (3)	C14—H14A	0.9900
C1—C2	1.529 (3)	C14—H14B	0.9900
C1—H1A	0.9900	C15—C16	1.541 (3)
C1—H1B	0.9900	C15—H15A	0.9900
C2—C3	1.543 (2)	C15—H15B	0.9900
C2—H2A	0.9900	C16—N6	1.464 (2)
C2—H2B	0.9900	C16—C17	1.533 (2)
C3—N1	1.461 (2)	C16—H16	1.0000
C3—C4	1.537 (2)	C17—O2	1.415 (2)
C3—H3	1.0000	C17—H17A	0.9900
C4—O1	1.415 (2)	C17—H17B	0.9900
C4—H4A	0.9900	C18—N7	1.322 (2)
C4—H4B	0.9900	C18—C19	1.414 (2)
C5—N2	1.320 (2)	C18—H18	0.9500
C5—C6	1.415 (2)	C19—C20	1.403 (2)
C5—H5	0.9500	C19—C22	1.443 (2)
C6—C7	1.407 (2)	C20—N6	1.348 (2)
C6—C9	1.447 (2)	C20—N8	1.377 (2)
C7—N1	1.349 (2)	C21—N8	1.463 (2)
C7—N3	1.372 (2)	C21—H21A	0.9800
C8—N3	1.468 (2)	C21—H21B	0.9800
C8—H8A	0.9800	C21—H21C	0.9800
C8—H8B	0.9800	C22—N10	1.358 (2)
C8—H8C	0.9800	C22—C23	1.399 (2)
C9—N5	1.351 (2)	C23—C24	1.378 (3)
C9—C10	1.400 (2)	C23—H23	0.9500
C10—C11	1.372 (3)	C24—N9	1.342 (2)
C10—H10	0.9500	C24—H24	0.9500
C11—N4	1.341 (3)	C25—N10	1.328 (2)
C11—H11	0.9500	C25—N9	1.338 (2)
C12—N5	1.329 (2)	C25—S2	1.7614 (19)
C12—N4	1.339 (2)	C26—S2	1.798 (2)
C12—S1	1.7594 (19)	C26—H26A	0.9800
C13—S1	1.799 (2)	C26—H26B	0.9800
C13—H13A	0.9800	C26—H26C	0.9800
C13—H13B	0.9800	N1—N2	1.383 (2)
C13—H13C	0.9800	N3—H3N	0.82 (2)
C14—O2	1.400 (3)	N6—N7	1.378 (2)
C14—C15	1.526 (3)	N8—H8N	0.83 (2)
			
O1—C1—C2	105.75 (16)	H15A—C15—H15B	109.1
O1—C1—H1A	110.6	N6—C16—C17	112.64 (14)
C2—C1—H1A	110.6	N6—C16—C15	112.50 (15)
O1—C1—H1B	110.6	C17—C16—C15	103.46 (15)
C2—C1—H1B	110.6	N6—C16—H16	109.4
H1A—C1—H1B	108.7	C17—C16—H16	109.4
C1—C2—C3	102.99 (15)	C15—C16—H16	109.4
C1—C2—H2A	111.2	O2—C17—C16	107.18 (15)
C3—C2—H2A	111.2	O2—C17—H17A	110.3
C1—C2—H2B	111.2	C16—C17—H17A	110.3
C3—C2—H2B	111.2	O2—C17—H17B	110.3
H2A—C2—H2B	109.1	C16—C17—H17B	110.3
N1—C3—C4	112.99 (14)	H17A—C17—H17B	108.5
N1—C3—C2	112.86 (14)	N7—C18—C19	112.69 (16)
C4—C3—C2	103.01 (15)	N7—C18—H18	123.7
N1—C3—H3	109.3	C19—C18—H18	123.7
C4—C3—H3	109.3	C20—C19—C18	103.83 (15)
C2—C3—H3	109.3	C20—C19—C22	126.39 (16)
O1—C4—C3	107.29 (15)	C18—C19—C22	129.77 (16)
O1—C4—H4A	110.3	N6—C20—N8	124.39 (16)
C3—C4—H4A	110.3	N6—C20—C19	106.85 (15)
O1—C4—H4B	110.3	N8—C20—C19	128.72 (16)
C3—C4—H4B	110.3	N8—C21—H21A	109.5
H4A—C4—H4B	108.5	N8—C21—H21B	109.5
N2—C5—C6	112.72 (15)	H21A—C21—H21B	109.5
N2—C5—H5	123.6	N8—C21—H21C	109.5
C6—C5—H5	123.6	H21A—C21—H21C	109.5
C7—C6—C5	103.87 (15)	H21B—C21—H21C	109.5
C7—C6—C9	126.33 (16)	N10—C22—C23	119.94 (16)
C5—C6—C9	129.80 (16)	N10—C22—C19	116.56 (15)
N1—C7—N3	124.83 (16)	C23—C22—C19	123.50 (16)
N1—C7—C6	106.75 (15)	C24—C23—C22	117.31 (17)
N3—C7—C6	128.40 (16)	C24—C23—H23	121.3
N3—C8—H8A	109.5	C22—C23—H23	121.3
N3—C8—H8B	109.5	N9—C24—C23	123.55 (17)
H8A—C8—H8B	109.5	N9—C24—H24	118.2
N3—C8—H8C	109.5	C23—C24—H24	118.2
H8A—C8—H8C	109.5	N10—C25—N9	127.40 (17)
H8B—C8—H8C	109.5	N10—C25—S2	118.83 (14)
N5—C9—C10	120.07 (16)	N9—C25—S2	113.77 (13)
N5—C9—C6	117.08 (15)	S2—C26—H26A	109.5
C10—C9—C6	122.86 (16)	S2—C26—H26B	109.5
C11—C10—C9	117.09 (17)	H26A—C26—H26B	109.5
C11—C10—H10	121.5	S2—C26—H26C	109.5
C9—C10—H10	121.5	H26A—C26—H26C	109.5
N4—C11—C10	123.96 (17)	H26B—C26—H26C	109.5
N4—C11—H11	118.0	C7—N1—N2	112.18 (14)
C10—C11—H11	118.0	C7—N1—C3	128.07 (15)
N5—C12—N4	127.39 (18)	N2—N1—C3	119.35 (14)
N5—C12—S1	119.05 (14)	C5—N2—N1	104.46 (14)
N4—C12—S1	113.57 (14)	C7—N3—C8	120.42 (15)
S1—C13—H13A	109.5	C7—N3—H3N	109.2 (17)
S1—C13—H13B	109.5	C8—N3—H3N	113.3 (17)
H13A—C13—H13B	109.5	C12—N4—C11	114.28 (16)
S1—C13—H13C	109.5	C12—N5—C9	117.16 (16)
H13A—C13—H13C	109.5	C20—N6—N7	112.26 (14)
H13B—C13—H13C	109.5	C20—N6—C16	127.71 (15)
O2—C14—C15	105.96 (16)	N7—N6—C16	119.47 (14)
O2—C14—H14A	110.5	C18—N7—N6	104.35 (14)
C15—C14—H14A	110.5	C20—N8—C21	121.13 (15)
O2—C14—H14B	110.5	C20—N8—H8N	107.4 (17)
C15—C14—H14B	110.5	C21—N8—H8N	113.2 (16)
H14A—C14—H14B	108.7	C25—N9—C24	114.62 (16)
C14—C15—C16	102.77 (16)	C25—N10—C22	117.17 (15)
C14—C15—H15A	111.2	C1—O1—C4	105.24 (15)
C16—C15—H15A	111.2	C14—O2—C17	106.04 (16)
C14—C15—H15B	111.2	C12—S1—C13	102.24 (9)
C16—C15—H15B	111.2	C25—S2—C26	102.10 (9)

### Hydrogen-bond geometry (Å, °)

**Table:**

*D*—H···*A*	*D*—H	H···*A*	*D*···*A*	*D*—H···*A*
N3—H3N···N5	0.82 (2)	2.16 (2)	2.828 (2)	139 (2)
N8—H8N···N10	0.83 (2)	2.13 (2)	2.820 (2)	140 (2)
